# Defensins and Sepsis

**DOI:** 10.1155/2014/180109

**Published:** 2014-08-19

**Authors:** Guo-Hao Xie, Qi-Xing Chen, Bao-Li Cheng, Xiang-Ming Fang

**Affiliations:** Department of Anesthesiology, the First Affiliated Hospital, Zhejiang University School of Medicine, Qingchun Road 79, Hangzhou 31003, China

## Abstract

Sepsis is a leading cause of mortality and morbidity in the critical illness. Multiple immune inflammatory processes take part in the pathogenesis of sepsis. Defensins are endogenous antimicrobial peptides with three disulphide bonds created by six cysteine residues. Besides the intrinsic microbicidal properties, defensins are active players which modulate both innate and adaptive immunity against various infections. Defensins can recruit neutrophils, enhance phagocytosis, chemoattract T cells and dendritic cells, promote complement activation, and induce IL-1*β* production and pyrotosis. Previous publications have documented that defensins play important roles in a series of immune inflammatory diseases including sepsis. This review aims to briefly summarize in vitro, in vivo, and genetic studies on defensins' effects as well as corresponding mechanisms within sepsis and highlights their promising findings which may be potential targets in future therapies of sepsis.

## 1. Introduction

Sepsis, severe sepsis, and septic shock represent a continuum of clinical syndromes which are common complications observed in patients with infection, trauma, and major surgeries [1–3]. These syndromes start with infection induced systemic inflammatory response syndrome (SIRS) and evolve to sepsis induced acute organ dysfunction and cardiovascular collapse. Epidemiology studies demonstrated that severe sepsis has a population prevalence of 300/100 000 in the United States and counts for 10–30% of the intensive care unit (ICU) patients [4–6]. And severe sepsis has already been acknowledged as the first cause of death in noncoronary ICUs with a high mortality rate of approximately 25–50% [7]. In the past one or two decades, steady progresses in treatment of sepsis have been made due to the advanced supportive care in ICU and the implementation of bundle therapies [7]. However, searching for specific remedies and reliable predictors within the pathophysiological mechanisms of sepsis is still the emphasis of today's studies [8, 9].

Defensins are classified as a subfamily of cationic antimicrobial peptides, which are major components of the human innate immunity. They are small endogenous peptides with three disulphide bonds created by six cysteine residues. Defensins are categorized into three subtypes, *α*-, *β*-, and *θ*-defensin, based on the spatial structure and the locations of three disulphide bonds within the peptide. In the past decade, cumulative evidences have suggested that defensins play an important role and may be a potential intervention target in sepsis. This review hereby will summarize in vitro, in vivo, and genetic studies on defensins' effects as well as corresponding mechanisms within sepsis and its sequential syndromes.

## 2. Antimicrobial Activities against Invading Pathogens in Sepsis

Defensins have broad spectrum antimicrobial activities against most pathogens in sepsis. The *α*-defensins are constitutively expressed in human neutrophils (human neutrophil peptides [HNP] 1–4) or intestinal Paneth cells (human defensin [HD] 5-6) [10–12]. They can inhibit a large variety of Gram-positive bacteria, Gram-negative bacteria, and some species of fungi and viruses [11].

The *β*-defensins are mainly distributed in the epithelial cells of the respiratory system, digestive system, and genitourinary system [10–12]. They can effectively kill a number of Gram-negative bacteria, such as* E. coli* and* P. aeruginosa*, Gram-positive bacteria, such as* S. aureus* and* Streptococcus pyogenes*, and* Candida albicans*. *β*-Defensin-3 even has bactericidal effect towards multiresistant* S. aureus* and vancomycin-resistant* Enterococcus* [11, 13].

The *θ*-defensins, which have a unique macrocyclic structure, are isolated from leukocytes from some species of monkey and have not been detected in humans [14]. They are also reported to have antimicrobial activity against a spectrum of pathogens including* E. coli*,* S. aureus,* and* C. albicans* [15]. Also, they are found to have protective effect in a mouse model from a lethal pulmonary infection by a mouse adapted strain of SARS-coronavirus [16].

The classic mechanism of defensins' bactericidal effect is the “pore formation” theory. These positively charged antimicrobial peptides target negatively charged bacterial membrane components, such as lipopolysaccharides, teichoic acids, or phospholipids. Then they form transmembrane pores, disrupt cell integrity, and lead to bacteria lysis [10, 11]. Recently, another mechanism has been reported that defensins kill bacteria by inhibiting the synthesis of bacterial cell wall through interaction with certain precursors such as lipid II [17].

Defensins' bactericidal effect can be limited by high salt concentration of local environment where they encounter with the pathogens [18, 19]. Also, the antimicrobial action appears to be regulated by the redox response, as *β*-defensin-1 become more potent after reduction of disulfide bridges by thioredoxin or a reducing environment [20, 21].

## 3. Modulators and Alarmins in Immune Inflammatory Response of Sepsis

Defensins are also reported to have modulating effects on both innate and adaptive immune response. It is well known that HNP1-3 participate in the host immune defense via multiple mechanisms, including enhancing macrophage phagocytosis, facilitating neutrophil recruitment, modulating complement activation, and chemoattracting immature T cells and dendritic cells [12, 22].

In vitro studies showed that *β*-defensins have potent chemotactic effects, leading to the recruitment and maturation of naive dendritic cells and memory T cells in the inflammatory sites and the triggering of specific immune response in the host [23]. As the endogenous ligand of TLR-4, *β*-defensins interact with TLR-4 of the immune cells and regulate the expression of inflammatory mediators via the NF-*κ*B pathway [18]. In vivo researches have revealed that the abnormal expression of *β*-defensins is associated with sepsis and various infectious diseases, as levels of *β*-defensins in both plasma and bronchoalveolar lavage fluid in patients with pulmonary infections are elevated [24–26], transcription of *β*-defensin-2 in leukocytes of severe septic patients is suppressed [27], expression of *β*-defensins in burn wound is reduced [28], and impaired expression of *β*-defensins is associated with inflammatory bowel diseases [29, 30]. In a mouse model of acute lung injury, Shu et al. expressed recombinant *β*-defensin-2 in lung tissue via recombinant adenovirus to study its protective effect against* P. aeruginosa* infection. Compared with control mice, they found considerably less* P. aeruginosa* in the transinfected lung tissue, as well as alleviated alveolar impairment, interstitial edema, and neutrophil infiltration [31, 32]. In subsequent studies, mice transinfected by adenovirus with or without *β*-defensin-2 genes received cecal ligation and puncture (CLP) twice to generate sepsis models. The impact of *β*-defensin-2 on the inflammatory response (e.g., the level of ICAM-1 expression), the severity of lung injury, and the sepsis outcome (7-day survival rate) were observed and evaluated. It was found that recombinant *β*-defensin-2 could downregulate the expression of ICAM-1 in lung tissue 24 h, 36 h, and 72 h after CLP and significantly raised the 7-day survival rate in sepsis mice [31, 33]. In the clinical setting, Olbrich et al. found preterm neonates had lower level of *β*-defensin-2 in cord blood when compared to term neonates [34]. And among these preterm neonates, lower *β*-defensin-2 level was associated with late-onset sepsis. These studies indicate that *β*-defensin-2 may play an important role in the immune inflammatory response in sepsis and might influence the outcome of sepsis.

Among the *θ*-defensins, rhesus macaque *θ*-defensin (RTD), which has six subtypes, has been extensively studied. Though not expressed in humans, RTDs were reported to significantly reduce levels of TNF-*α*, IL-1*β*, IL-6, IL-8, MIP1, and so on, in human peripheral blood leukocytes that are preincubated with various toll-like receptor agonists [35]. Furthermore, in vivo study showed that subcutaneously administration of 5 mg/kg RTD-1 could improve the survival rate and suppress the levels of a number of inflammatory cytokines and chemokines in two sepsis mouse models (received either intraperitoneal injection of* E. coli* or CLP). Although detailed mechanisms of the protective effect of RTD-1 have not been illuminated, the authors suggested that the interaction between RTD-1 and leukocyte is the critical determinant of TNF-*α* blockade [35]. The latter is a major proinflammatory cytokine and influences the consequent inflammatory cascades. These results indicate that *θ*-defensins may be a potential immune adjuvant in the treatment of sepsis, though they are not expressed in human.

In sepsis and other inflammatory disorders, defensins are among a group of rapidly-released host endogenous molecules, which are capable of both recruiting and activating APCs and are also termed the alarmins. Recently, in vitro studies have shown that alarmin HNP1-3 have the ability to boost host inflammatory response by promoting macrophage IL-1*β* production and pyroptosis via purinergic P2X7 receptor [36]. However, this effect is a double-edged sword in sepsis since it can promote pathogen elimination as well as mediate organ dysfunction such as acute lung injury [22, 37].

## 4. Genetic Polymorphisms and Sepsis Susceptibility

In molecular genetics and molecular biology, knock-out animal model is one of the most convincing means to determine the role of a specific molecule in the physiopathology of a certain disease. However, as members of the defensin family have overlapped biological functions, the function of the knock-out gene in animal models may probably be compensated by other defensins. Since the gene cluster coding for the entire defensin family cannot be fully knocked out using the present techniques of molecular biology and genetics as well as human defensins lack of absolute animal analogues, genetic association analysis is a good alternative that can effectively explore the relationship between genetic polymorphism and sepsis.

In normal peripheral blood cells, mRNA levels of both *β*-defensin-1 and *β*-defensin-2 raise remarkably when stimulated by LPS or* P. aeruginosa* [23]. However, the upregulation of *β*-defensin-1 and *β*-defensin-2 varies among individuals, resulting in interindividual differences in host defense capacity and hence influencing the clinical progression of sepsis. Previous studies showed that single nucleotide polymorphism (SNP) of *β*-defensin-1 gene (DEFB1) correlates with chronic obstructive pulmonary disease, asthma, genetic allergy, HIV infection, and pseudomonas species infection in oral mucosa [38–42]. Since sepsis is a multifactorial disease caused by both environmental factors (pathogenic microbes) and host factors (comorbidities and genetic background), its occurrence and outcome are influenced with individual genetic background [43]. Chen et al. selected 5 SNPs in the promote region of DEFB-1 (–1816A/G, –390A/T, –52A/G, –44C/G, and –20A/G) and one in its extron (1654G/A) as candidate loci and studied 211 patients with severe sepsis and 157 healthy controls [44]. Distribution of alleles, gene types, and haplotypes associating with these loci were studied and compared between septic patients and controls, as well as between survivals and victims of severe sepsis. Association analysis, logistic regression, and linkage disequilibrium study showed that –44G allele was closely related with susceptibility to severe sepsis and poor outcome. And severe septic patients with haplotype –20G/–44G/–52G had even poorer outcome, while individuals with haplotype –20A/–44C/–52G were less susceptible to severe sepsis. The reason why –44C/G is correlated with the occurrence and outcome of severe sepsis may attribute to the following points. It located in the 5′ untranslated region of DEFB1 and its polymorphism may result in changes in the space conformation of mRNA, which would alter the stability of mRNA and the efficiency of translation. And its impact on the protein function is more significant than nonsynonymous SNP in coding region [45], as the quantity of protein would change dramatically. However, as any other genetic association analysis, DEFB1 −44C/G may be only a surface marker of some unknown real genetic marker of sepsis in linkage disequilibrium. Although these hypothesis need to be proved by further researches, the above-mentioned study indicated that *β*-defensin-1 might be an influential factor in the process of immune defense and inflammation regulation in sepsis, and the locus of −44C/G may be an important genetic warning indicator of susceptibility to severe sepsis and its outcomes.

Copy number variation (CNV) is a kind of genetic polymorphism that accounts for approximately 12% of human genomic DNA. It refers to a large-scale duplication or deletion of certain DNA sections, which causes a variation in the number of copies of one or more genes. Previous publications reported that CNV is present in *β*-defensin-2 gene (DEFB4), *β*-defensin-3 gene (DEFB103), *β*-defensin-4 gene (DEFB104), *α*-defensin-1 gene (DEFA1), and *α*-defensin-3 gene (DEFA3) [18, 46–48]. And copy number of DEFB4 has a positive correlation with its mRNA level [35, 45]. Recently, Chen et al. screened 179 severe sepsis and 233 healthy controls for DEFA1 and DEFA3 [49]. An average DEFA1/DEFA3 copy number of 7 per genome was observed in the studied population, with a range of 2 to 15. The authors found that patients with high copy number of DEFA1/DEFA3 were predisposed to severe sepsis and tended to have lower level of plasma HNP1-3 as well as cytokines such as TNF-*α*, IL-6, and IL-10. They further validated their findings in an independent cohort. These results indicated that CNVs in the defensin gene may be potential genetic markers for identifying high risk patients or providing individual treatment in sepsis.

## 5. Perspective 

Defensins are emerging therapeutic molecules against pathogens in sepsis because of their broad spectrum antimicrobial properties. In the past decade or two, a number of potent and salt insensitive defensins and their analogs have been screened, structurally modified, and synthesized. However, most of these studies are performed in vitro and not much is known about the in vivo roles of these molecules. In fact, chemoattracting and immunomodulating effects make defensins a double-edged sword in the pathogenesis of sepsis, which leads to facilitation of pathogen clearance as well as exacerbation of inflammation and injury of self-tissues. Recently, several investigations showed that the chemoattractant and antimicrobial activities of defensins could be separated, which shed light on the design of defensin-derived pharmaceuticals [50]. In addition, genetic studies help identify high risk patients with susceptibility to sepsis or its adverse outcome, which provides foundation for future individualized sepsis treatments that are targeting defensins.
